# Cerebellar Dysfunction as a Source of Dystonic Phenotypes in Mice

**DOI:** 10.1007/s12311-022-01441-0

**Published:** 2022-07-12

**Authors:** Amanda M. Brown, Meike E. van der Heijden, H. A. Jinnah, Roy V. Sillitoe

**Affiliations:** 1 Department of Pathology & Immunology, Baylor College of Medicine, Houston, TX USA; 2 Jan and Dan Duncan Neurological Research Institute at Texas Children’s Hospital, Houston, TX 77030 USA; 3 Departments of Neurology, Human Genetics and Pediatrics, Emory University School of Medicine, Atlanta, GA 30322 USA; 4 Department of Neuroscience, Baylor College of Medicine, Houston, TX USA; 5 Department of Pediatrics, Baylor College of Medicine, Houston, TX USA; 6 Development, Disease Models & Therapeutics Graduate Program, Baylor College of Medicine, Houston, TX USA

**Keywords:** Dystonia, Purkinje cells, Cerebellar nuclei, Genetic mouse models, Electrophysiology

## Abstract

There is now a substantial amount of compelling evidence demonstrating that the cerebellum may be a central locus in dystonia pathogenesis. Studies using spontaneous genetic mutations in rats and mice, engineered genetic alleles in mice, shRNA knockdown in mice, and conditional genetic silencing of fast neurotransmission in mice have all uncovered a common set of behavioral and electrophysiological defects that point to cerebellar cortical and cerebellar nuclei dysfunction as a source of dystonic phenotypes. Here, we revisit the *Ptf1a*^*Cre/*+^*;Vglut2*^*flox/flox*^ mutant mouse to define fundamental phenotypes and measures that are valuable for testing the cellular, circuit, and behavioral mechanisms that drive dystonia. In this model, excitatory neurotransmission from climbing fibers is genetically eliminated and, as a consequence, Purkinje cell and cerebellar nuclei firing are altered in vivo, with a prominent and lasting irregular burst pattern of spike activity in cerebellar nuclei neurons. The resulting impact on behavior is that the mice have developmental abnormalities, including twisting of the limbs and torso. These behaviors continue into adulthood along with a tremor, which can be measured with a tremor monitor or EMG. Importantly, expression of dystonic behavior is reduced upon cerebellar-targeted deep brain stimulation. The presence of specific combinations of disease-like features and therapeutic responses could reveal the causative mechanisms of different types of dystonia and related conditions. Ultimately, an emerging theme places cerebellar dysfunction at the center of a broader dystonia brain network.

## Introduction

Dystonia is a neurological disease that causes muscles to work against, rather than with one another [1]. Dystonia names both a symptom and a disease, which can be acquired, genetic, or idiopathic. The main symptoms can be mild or transient, appearing only under conditions of exertion or fatigue, or severe and constant enough to destroy a livelihood or make walking impossible. Dystonia has recently been redefined as a “movement disorder characterized by sustained or intermittent muscle contractions causing abnormal, often repetitive, movements, postures, or both” [2], making the presence of abnormal muscle contractions the primary indicator of dystonia. Over-contraction of the affected muscles is the main component of this abnormal muscle activity, however in some cases, agonist and antagonist muscles erroneously co-contract [3]. The involuntary muscle behavior can result in painful episodes that affect any muscle(s) in the body, causing blepharospasm in the eyelids (a frequent result of antipsychotic drugs [4]), writer’s cramp [5], or inherited torsion dystonia to an extent that makes daily behaviors impossible [6]. Although dystonia is considered the third most common movement disorder, its true prevalence is challenging to accurately estimate because it can be comorbid with other relatively common disorders or rare motor diseases including tremor, Parkinson’s disease, Huntington’s disease, stroke, epilepsy, and ataxia [7, 8]. Moreover, in many instances, milder cases are not reported [9, 10]. Dystonia is also a clinical sign that can be the presenting or prominent manifestation of many neurodegenerative and neurometabolic disorders. Despite the wide range of manifestations and causes, dystonia is thought to involve faulty function in a network [11] that includes the cerebral cortex, basal ganglia, thalamus, brainstem, and cerebellum [12–16]. Therefore, defects within a critical motor network may be responsible for dystonia.

Great efforts have been made to determine the relationship between possible dystonia etiologies and the spectrum of known clinical characteristics of the disease. Many cases of isolated dystonia, where dystonia is not comorbid with other movement disorders, are thought to be genetic in origin. Among the most common genes with mutations that cause dystonia are *TOR1A*, *GNAL*, *SGCE*, and *THAP1* [1, 17, 18]. While in many studies knocking out the dystonia-causing genes only caused mild or general motor defects, other manipulations such as shRNA knockdown induced a powerful phenotype [19]. Still, the genetic models, particularly manipulations of *Tor1a* in mice, have paved the way for testing the circuits [16, 20] and anatomical alterations in dystonia [21]. The cerebellum is now identified as a key target in dystonia [22]. These studies are in accordance with previous work from a genetically dystonic (dt) rat model, which indicated cerebellar circuit abnormalities in this disease [23]. More recent work examining the pharmacological application of ouabain into the cerebellum and basal ganglia further implicated cerebellar dysfunction as a driver of dystonia [24]. These studies were supported by the demonstration of irregular Purkinje cell function after ouabain infusion [25] and shRNA knockdown of the α3-containing sodium pumps, which are heavily expressed in Purkinje cells and are the targets of ouabain [26]. Similarly, Purkinje cell firing was also irregular in an induced model of DYT1 dystonia that also utilized shRNA to knock down *Tor1a* in the cerebellum [19]. Mechanistic insights into the role of the cerebellum in dystonia have therefore been acquired through multiple animal model systems and using different manipulation strategies in vivo.

A key issue that has been under scrutiny is the exact defects in the cerebellum that are responsible for initiating the dystonia. Much attention has been placed on two primary cell groups, the Purkinje cells in the cerebellar cortex and the neurons in the cerebellar nuclei. Normally, both types of cells fire at relatively high rates, often exceeding ~50 Hz. In addition, although their in vivo firing pattern is not tonic, both cell types fire with robust and continuous activity with minimal pausing in healthy awake animals (although brief pauses are indeed a key feature that may be required for precise control of movement kinematics [27]). In dystonia, a more pronounced number of pauses, in this case, dramatic enough for the remaining action potentials to occur in interspersed “bursts” or erratic firing, may drive abnormal behavior. This hypothesis is supported by results in the dt rat model [28], the *Atcay*^*ji−hes*^ mouse model of dystonia [29], the ouabain infusion mouse model [25], and the laminin mutant, *lamb1t* [30]. To better define the neural circuits and cellular mechanisms that might generate the bursts, we devised a conditional genetic mouse model to specifically target cerebellar function in vivo. Toward this, we generated the *Ptf1a*^*Cre/*+^*;Vglut2*^*flox/flox*^ mice [31]. These mice were designed to selectively block fast neuronal communication from climbing fibers to Purkinje cells. Due to the direct structural and functional connectivity with the cerebellar nuclei downstream, defects in Purkinje cell firing activity were predicted to instigate erratic responses in the cerebellar nuclei neurons of *Ptf1a*^*Cre/*+^*;Vglut2*^*flox/flox*^ mutant mice [31].

To generate the mice, we crossed male mice that were heterozygous carriers of the *Ptf1a*^*Cre*^ allele [32] and homozygous for a *LoxP*-flanked glutamatergic vesicular transporter 2 allele, *Vglut2*^*flox*^ (JAX #012898) [33], to female mice that were homozygous for *Vglut2*^*flox*^. The resulting *Ptf1a*^*Cre/*+^*;Vglut2*^*flox/flox*^ offspring had a conditional deletion of the *Vglut2* allele that is specific for the *Ptf1a* lineage (achieved by driving the Cre). As a consequence, the loading of glutamate into presynaptic vesicles during fast neurotransmission is genetically blocked, which results in the precise elimination of neurotransmission at glutamatergic synapses only within the *Ptf1a*-expressing neurons [31]. Importantly, most neurons in the *Ptf1a* lineage are inhibitory and therefore are not affected by the removal of *Vglut2*. However, the inferior olivary neurons in the brain do express both *Ptf1a* and *Vglut2* and, ultimately, the olivary climbing fiber projections that terminate upon the dendrites of Purkinje cells in the molecular layer of the cerebellar cortex no longer express the VGLUT2 protein at their synapses.

Here, we describe the application of these mice to behavior, electrophysiology, and deep brain stimulation and argue that mouse models of cerebellar function hold critical insights for understanding dystonia pathogenesis and treatment options. The specificity of the approach not only allows for the dissection of cerebellar mechanisms in dystonia, but also provides a framework for disentangling how the cerebellum might contribute to a number of motor disorders, including ataxia, tremor, and seizures [34–36]. We discuss how we have arrived at the current circuit and network model of dystonia. Notably, previous seminal findings established dystonia as a neurological disease and placed the basal ganglia at the center of a critical motor network in hemidystonia [37]. Such studies have allowed us to expand upon the many clinical observations and test how brain regions that were previously speculated to contribute to associated conditions such as athetosis, namely the cerebellum [38], might interact with the basal ganglia to drive dystonia across different forms of the disease [24, 39]. The current discussion centers around the design of animal models, data collection approaches, analysis techniques, and the use of preclinical therapeutic tools to deepen our understanding of dystonia.

## Materials and Approach

The experimental approaches and analyses presented here were described in detail in White and Sillitoe, 2017 [31]. In addition, please refer to our recent work for further details [35, 36, 40]. Images were drawn and imported into Adobe Illustrator or created directly in Adobe Illustrator. Some image panels were contrast-corrected in Adobe Photoshop before the final figures were assembled.

## Methods and Results

### Twisting Postures of the Limbs and Torso, Hyperextension of the Neck, Tail, and Digits

**Fig. 1:**
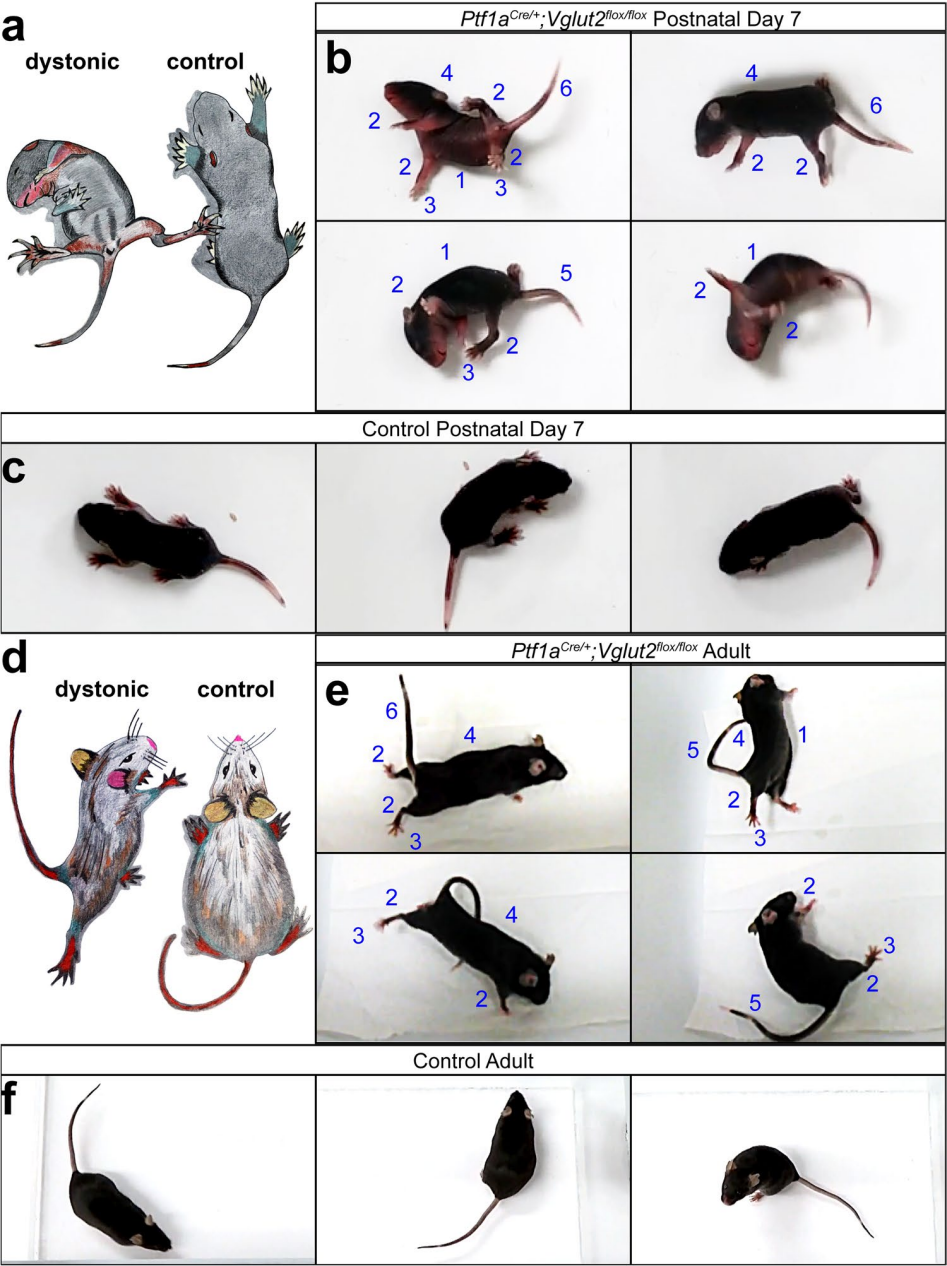
Dystonic behavior in developing and adult *Ptf1a*^*Cre/*+^*;Vglut2*^*flox/flox*^ mice. **a** Schematic illustration depicting the overall dystonic behaviors observed in developing *Ptf1a*^*Cre/*+^*;Vglut2*^*flox/flox*^ mice. **b** The mutant pups exhibit a number of dystonic features. Twisting of the limbs and torso (1), hyperextension of the limbs (2), splaying of all five digits (3), hyperflexion of the neck and back muscles that causes upward flexion of the torso (4), tail kink (5), tail exhibits powerful hyperextension and stiffness (6). **c** Frames from a video recording of a control pup demonstrating typical behavior. **d** Schematic illustrating the dystonic movements observed in adult *Ptf1a*^*Cre/*+^*;Vglut2*^*flox/flox*^ mice. **e** Frames from a *Ptf1a*^*Cre/*+^*;Vglut2*^*flox/flox*^ adult demonstrating dystonic behavior. See (b) for the different behaviors that are indicated by labels 1–6). **f** Frames from a video recording of an adult control mouse demonstrating typical behavior

Twisting of the limbs and torso is a primary symptom in severe forms of dystonia. These specific features of the disease have been difficult to replicate in mouse models of common genetic mutations associated with dystonia in humans. The *Ptf1a*^*Cre/*+^*;Vglut2*^*flox/flox*^ mutant has proven to be especially useful for studying these severe dystonic symptoms. These include twisting of the torso (Fig. 1a–f) and hyperextension of the limbs (Fig. 1a–f), which are obvious starting during the first postnatal week (although the mice are affected before that) [31]. It is also clear that the digits, particularly of the hindlimbs, exhibit hyperextension and splaying of all five digits (Fig. 1b,e). Importantly, in addition to the hyperflexion of the neck and back muscles that causes upward flexion of the torso, the tail exhibits powerful hyperextension and stiffness (Fig. 1b,e). These phenotypes can all co-occur in the more severe periods that mimic an attack (Fig. 1), while at other times they can occur in subsets of body parts. It is important to note that even when the twisting and hyperextension behaviors are less severe, the mice are compromised in their motor function, which is observed as stiff gait and jerky motion [31].

### EMG, Co-contractions and Cross Correlations, and Sustained Over-contractions in Dystonia

**Fig. 2:**
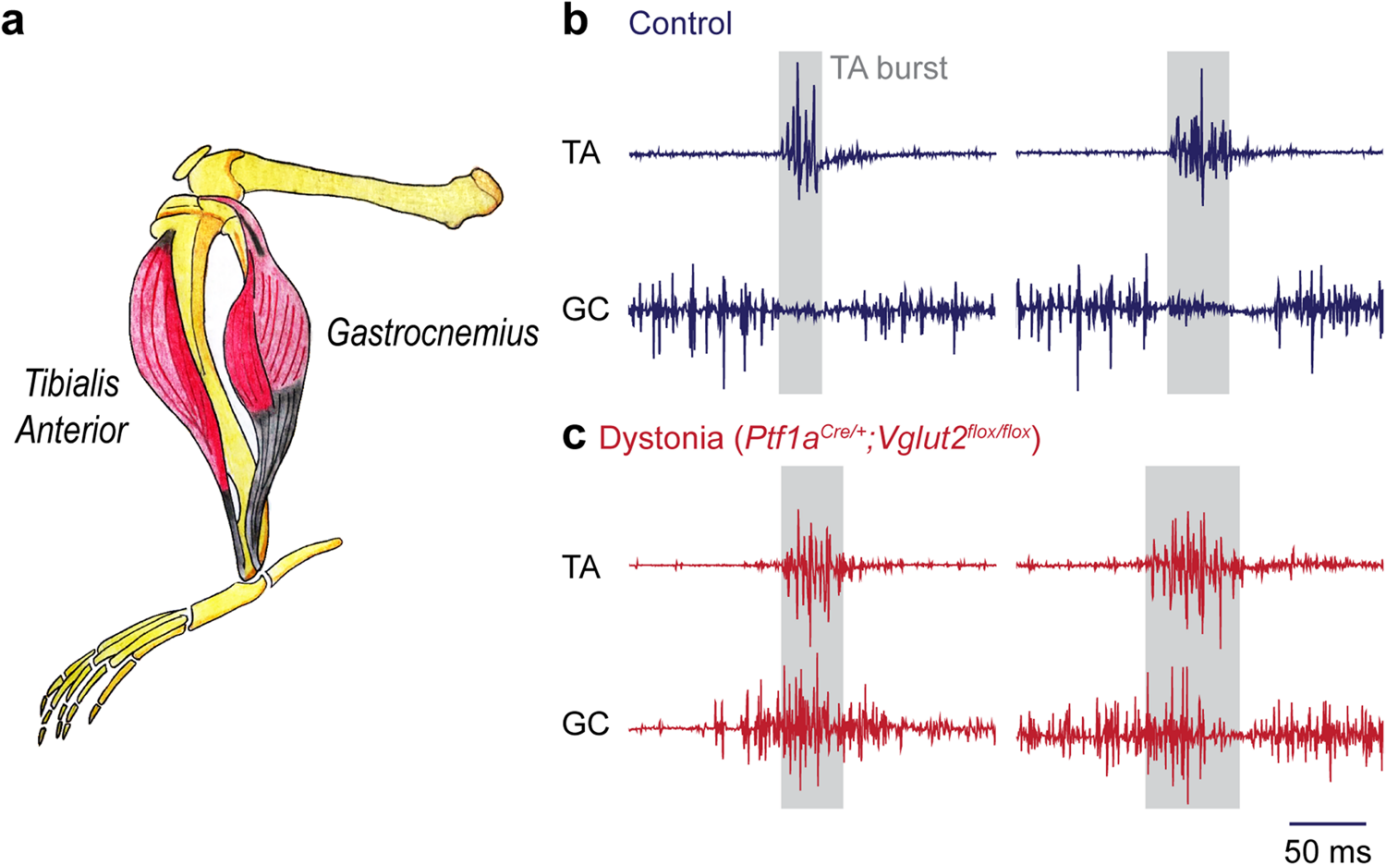
Muscle co-contractions and over-contractions in dystonic mice. **a** EMG signal for the analysis of dystonic phenotypes can be efficiently collected from the tibialis anterior (TA) and gastrocnemius (GC) muscles in the hindlimb. The schematic illustrates the anatomical location of these two muscles in the adult mouse leg. **b** In control mice, the TA and GC fire out of phase with one another during normal locomotion. **c** In *Ptf1a*^*Cre/*+^*;Vglut2*^*flox/*^^*flox*^ mice, the TA and GC fire at the same time, which is indicative of a co-contraction. In addition, note that the GC remains active for longer periods than the control GC, with more spikes occurring per active period

The twisting of the torso and limbs in the *Ptf1a*^*Cre/*+^*;Vglut2*^*flox/flox*^ mutants is accompanied by muscle physiology changes that reflect these abnormal movements. We are able to detect specific muscle defects using electromyography (EMG). We implant EMG recording electrodes into the tibialis anterior (TA) and the gastrocnemius muscles (GC) (Fig. 2a). During normal locomotion, proper kinematics about the leg joints requires that these two muscles fire out of phase with one another in order to have proper limb motion (Fig. 2b). Consistent with the reported co-contraction of agonist and antagonist muscles in dystonia, simultaneous EMG recordings of the TA and GC show co-contractions in *Ptf1a*^*Cre/*+^*;Vglut2*^*flox/flox*^ mice (Fig. 2c). Cross-correlations can then be used to quantitatively examine the extent of simultaneous muscle contractions during a given recording period [41]. Dystonia is commonly associated with over-contractions of the muscles. The *Ptf1a*^*Cre/*+^*;Vglut2*^*flox/flox*^ mutant mice indeed also express this phenotype (Fig. 2c). Therefore, the complex postural and kinetic defects that are observed in dystonia reflect an underlying combination of muscle mis-contractions that impact several features of movement.

### Tremor as a Prominent Feature Coexisting with Dystonia

**Fig. 3:**
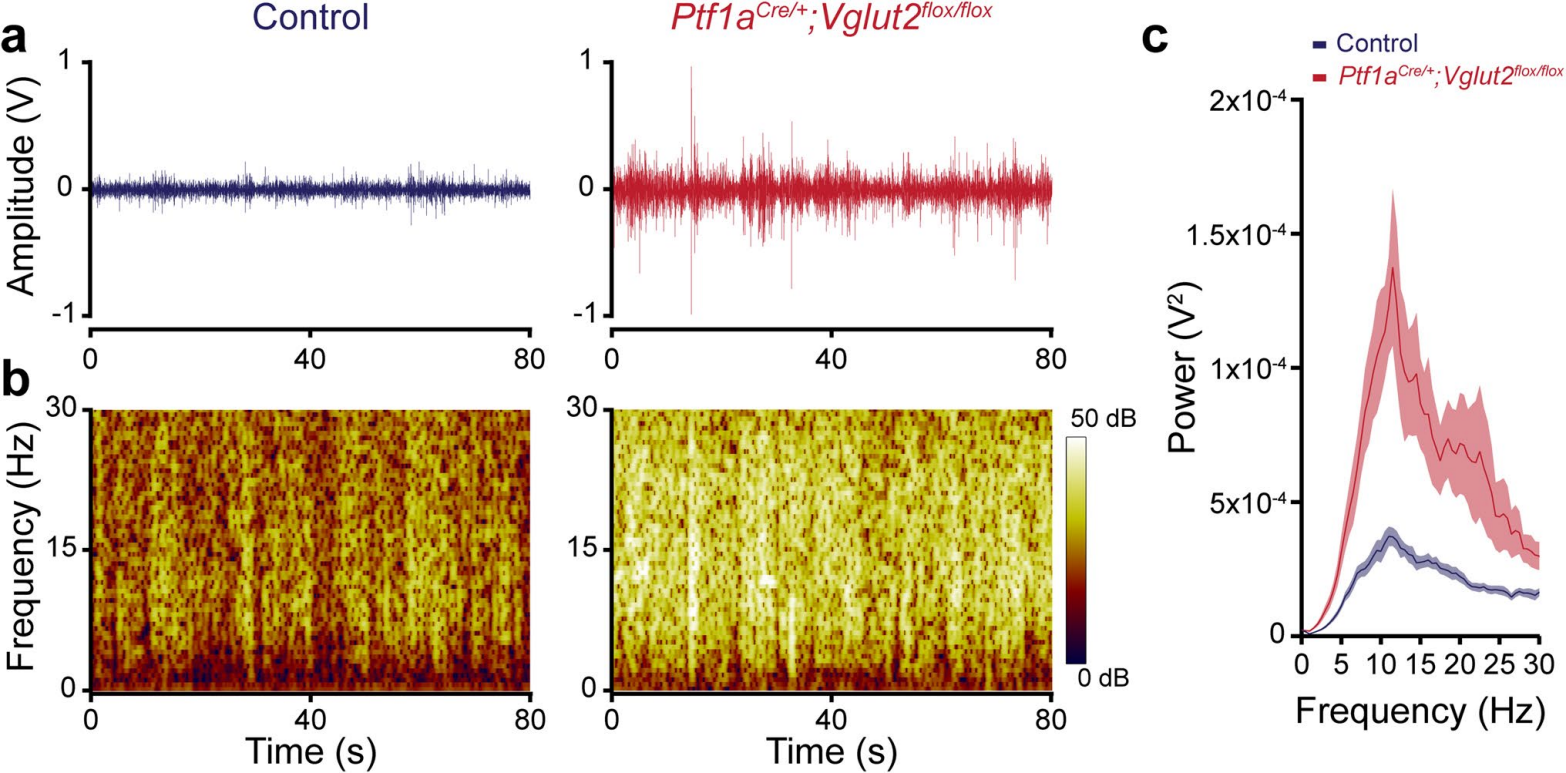
Dystonia with tremor. *Ptf1a*^*Cre/*+^*;Vglut2*^*flox/flox*^ mice have a tremor in addition to dystonia. **a** Example raw tremor recording traces detected with a tremor monitor for a control (left) and *Ptf1a*^*Cre/*+^*;Vglut2*^*flox/flox*^ mouse (right). **b** Example continuous power spectrums from the raw traces in (a). Different frequencies of tremor may be detected with different powers depending on the specific behavior, or in some cases composite set of dystonic behaviors, that are occurring at a given moment. Heat scale = 0 to 50 dB. **c** Power spectrum analysis of control (*N* = 10) and *Ptf1a*^*Cre/*+^*;Vglut2*^*flox/flox*^ (*N* = 6) mice. An ~10 Hz tremor can be extracted from the *Ptf1a*^*Cre/*+^*;Vglut2*^*flox*^^*/flox*^ mutants. Solid line = mean. Shaded region = standard error of the mean

It is estimated that upwards of 50% of individuals with dystonia also have tremors [8]. At the behavioral level, tremor is characterized by oscillatory and repetitive movements that typically affect the limbs and head. Tremors can be derived from central and peripheral mechanisms [42]. Within the central nervous system, several key areas including the cerebellum and thalamus are thought to be regions that are capable of inducing tremors [43]. In the cerebellum, interactions between the inferior olive and Purkinje cells are one potentially powerful source for initiating oscillatory neural activity that facilitates neuronal synchrony to drive muscle oscillations [35, 44]. In the *Ptf1a*^*Cre/*+^*;Vglut2*^*flox/flox*^ mutants, which lack inferior olive to Purkinje cell communication, an ~10 Hz tremor accompanies the severe dystonia [31]. In animal models, tremorgenic oscillations can be recorded using accelerometer-based devices [35, 45] (Fig. 3a) or using EMG [35]. A power spectrum analysis performed in Spike2 (Cambridge Electronic Design, Cambridge, UK) or equivalent software can be used to apply a fast Fourier transform (FFT) on the signal to determine the power of its frequency components, allowing the comparison of the strength of tremor at specific frequencies or within bands of frequencies (see [35], Fig. 3b,c). Such recording approaches are also sensitive enough to examine the contribution of tremor in developmental models of dystonia [46] and in dystonia models that mimic the genetic bases, although have more subtle phenotypes, as in the *Thap1* mice [40]. We argue that in animal models of dystonia, tremor is a valuable quantitative behavior to examine even if other phenotypes such as severe twisting postures and distinct episodes or “attacks” are subtle or absent.

### Altered Firing of Purkinje Cells and Cerebellar Nuclei Neurons in Dystonia

**Fig. 4:**
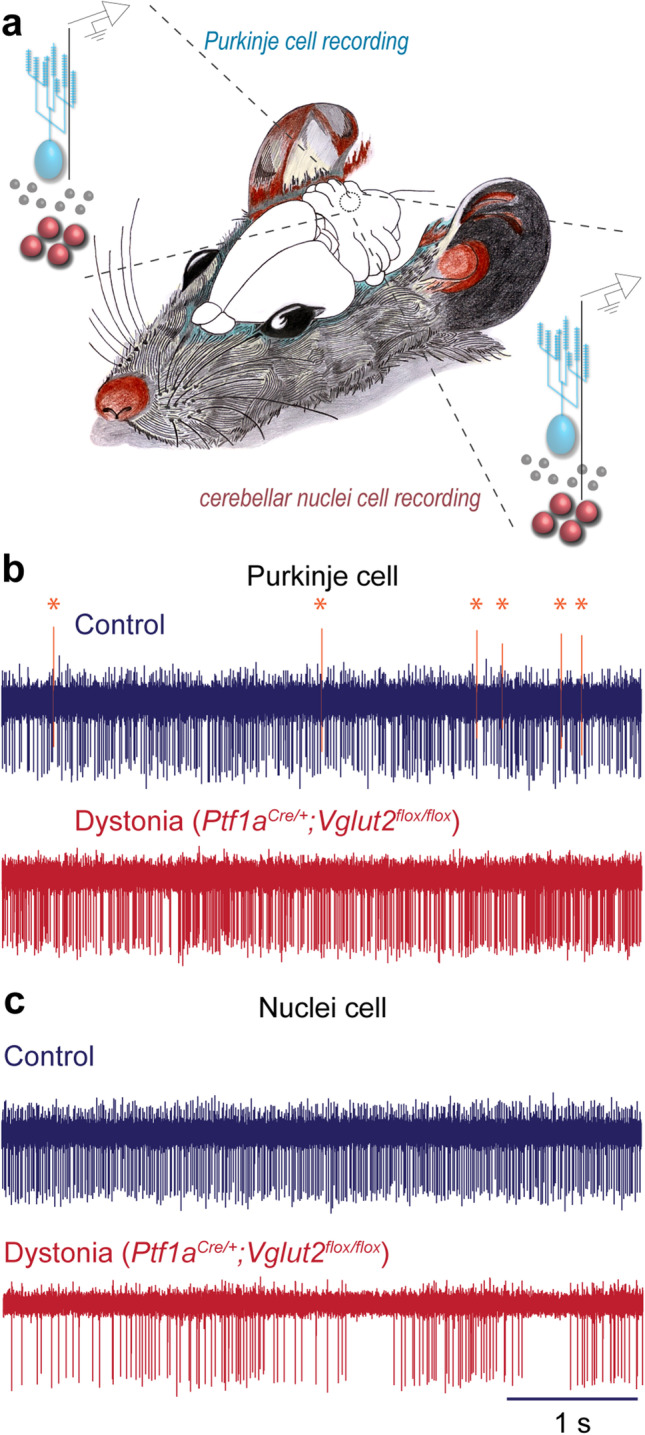
Abnormal cerebellar activity in mice with dystonic behavior. **a** Schematic illustrating the recording set up for in vivo electrophysiology. Single-unit extracellular signals are recorded from Purkinje cells and cerebellar nuclear neurons in awake mice. **b** Purkinje cells lack complex spikes (labeled with asterisks and orange in the control) in the *Ptf1a*^*Cre/*+^*;Vglut2*^*flox/flox*^ mutant mice. **c** Cerebellar nuclear neurons have a bursty pattern of firing in the dystonic mutant mice

Cerebellar Purkinje cells and nuclei neurons fire at relatively high rates in behaving animals. In mice, Purkinje cell simple spikes and nuclear neurons fire on average at ~65–70 Hz [35, 47] (Fig. 4a,b). Purkinje cell complex spikes, which are initiated by climbing fiber input, occur at ~1 Hz. Although firing rate can be affected in different models of cerebellar disease, for example ataxia [48], there is consistent evidence that firing pattern is primarily affected in dystonia. By measuring the coefficient of variance (CV) of the interspike intervals or the spike-to-spike variability (CV2; [49]), several studies in mouse and rat models report significant changes in Purkinje cell and nuclear neuron behavior in a manner that results in bursts of spike activity [19, 25, 28–30, 50]. In accordance with these studies, the *Ptf1a*^*Cre/*+^*;Vglut2*^*flox/flox*^ mutant mice also exhibit burst firing of the cerebellar nuclei neurons [31] (Fig. 4c). Similar to complete removal of the cerebellum or more select lesions of the nuclei in the genetically dystonic rat [51], lidocaine infusion into the interposed cerebellar nuclei reversibly blocks the twisting postures and tremor in the *Ptf1a*^*Cre/*+^*;Vglut2*^*flox/flox*^ mutant mice [31]. Intriguingly, this burst pattern of cerebellar nuclei neuron activity changes over development despite the animals appearing dystonic from early postnatal days. At juvenile ages, both *Ptf1a*^*Cre/*+^*;Vglut2*^*flox/flox*^ mutant mice and control mice have a relatively high cerebellar nuclei neuron CV compared to that of control animals in adulthood. However, at this time point, *Ptf1a*^*Cre/*+^*;Vglut2*^*flox/flox*^ nuclei neuron activity is of higher frequency to that of similarly aged control animals. Since at this age both mutant and control animals exhibit burst activity from cerebellar nuclei cells, this increase in frequency reflects either an increased number of bursts or an increased number of spikes within a burst of activity compared to that of control mice. This irregular activity of the cerebellar nuclei appears to be essential to the maintenance of the dystonic phenotype. While most Purkinje cells in *Ptf1a*^*Cre/*+^*;Vglut2*^*flox/flox*^ mice do not fire complex spikes at both juvenile and adult time points (Fig. 4b), Purkinje cell simple spike activity is slower and is more tonic in juvenile *Ptf1a*^*Cre/*+^*;Vglut2*^*flox/flox*^ mice compared to that of similarly aged controls. These differences in simple spike frequency and pattern disappear by adulthood, leaving the irregular firing of the cerebellar nuclei as the predominant aberrant signal that is retained throughout development and communicated from the cerebellum. These studies support the hypothesis that abnormal burst firing in the cerebellum is central to dystonic behavior.

### Cerebellar Deep Brain Stimulation Normalizes Abnormal Movement in Dystonia

**Fig. 5:**
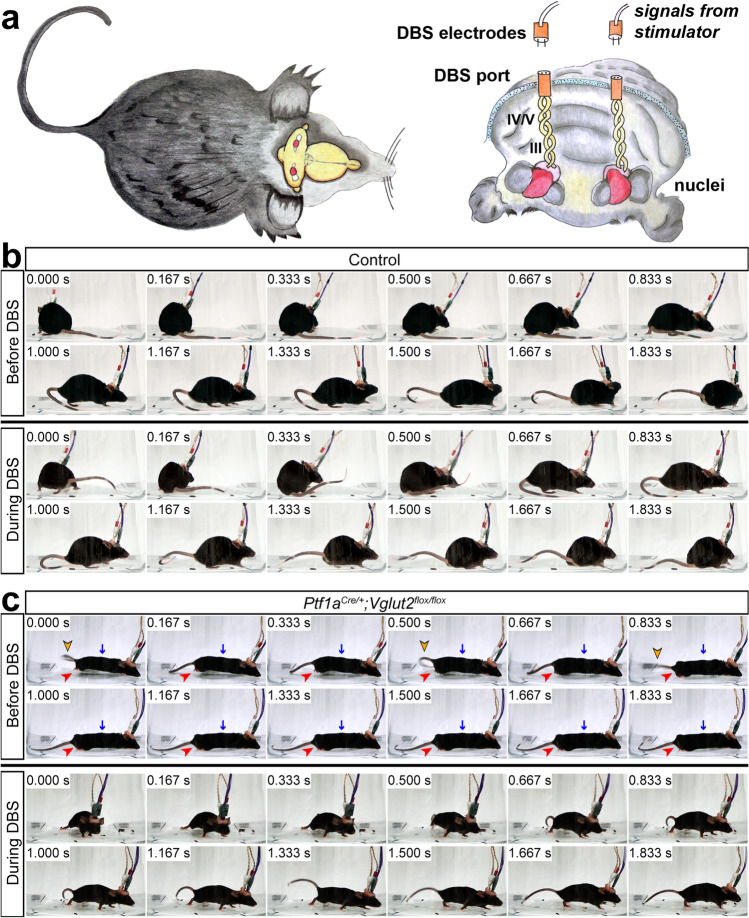
Cerebellar deep brain stimulation reduces dystonic postures and improves mobility. **a** Schematics illustrating our approach of targeting deep brain stimulation (DBS) to the interposed nucleus (pink). The schematic on the left shows the mouse from a top view with the brain visualized. The schematic on the right shows a front view of the cerebellum with the anterior-most structures cut away to illustrate the targeting of bipolar stimulating electrodes into the interposed nuclei. The electrode holder and port are shown in orange and the bipolar electrodes are colored yellow. **b** DBS in controls does not impact normal movement. **c** DBS restores mobility in *Ptf1a*^*Cre/*+^*;Vglut2*^*flox/flox*^ mice. Note that abnormal body posture (blue arrow), hyperflexion of the tail (yellow arrowhead), and altered limb movement (red arrowhead) are eliminated with DBS

The cerebellar nuclei are comprised of three pairs of sub-nuclei. From medial to lateral they are the fastigial, interposed (globose and emboliform in primates), and dentate nuclei. In regards to dystonia, the interposed is an interesting area because of its role in limb movements and ongoing motion [52–54]. Accordingly, the mouse cerebellar interposed contains projection neurons that connect to a number of motor-associated spinal cord and brainstem regions and collateralize to motor areas of the thalamus [54–56]. Ablation of a subset of glutamatergic nuclei neurons that express Urocortin3 in the anterior interposed (IntA) nucleus disrupts accurate limb positioning and timing in a reach-to-grasp task and during locomotion [54]. Chemogenetic silencing of excitatory neurons that project ipsilaterally to the cervical regions of the spinal cord also disrupts reach success in mice [56]. In addition, closed-loop manipulation of the IntA disrupts the reach endpoint in real-time [52]. In addition to sculpting reach and gait kinematics, the interposed nucleus also mediates conditioned eyelid responses and is responsive to tactile stimulation [57–59]. Blocking or modifying abnormal interposed output in the *Ptf1a*^*Cre/*+^*;Vglut2*^*flox/flox*^ mutant mice could therefore engage these circuit mechanisms [31]. Deep brain stimulation directed to the interposed nuclei recovered mobility in *Ptf1a*^*Cre/*+^*;Vglut2*^*flox/flox*^ mice [31] (Fig. 5a–c). Impressively, deep brain stimulation of the interposed is also beneficial in a mouse model of tremor [35] and in the *Car8*^*wdl/wdl*^ mutant, which displays ataxia, tremor, and dystonia [36].

## Discussion

There are a growing number of models that have immense utility in experiments designed to better understand dystonia. Among these models are approaches that use in vivo genetics in mice and rats, shRNA knockdown, and brain tissue injection of pharmacological compounds. Although not all models display all characteristics of dystonia, and indeed the severe twisting postures have been central to many debates about what makes a “good” model for dystonia, every model has provided its own important contributions to examining dystonia pathophysiology. For example, the genetically dystonic dt rat gave the initial impression of cerebellar activity in rodents with dystonia [23] and the *Dyt1* null mice were key in uncovering a network impact [16], whereas knockin of a mutant *Dyt1* sequence revealed circuit, behavioral, and developmental effects in dystonia [20], phenotypes which were interestingly also seen in a forebrain knockout of *Dyt1* [60]. The *Atcay*^*ji−hes*^ mouse taught us about the high-stepping gait [29] and ouabain infusion into the cerebellum revealed the dramatic twisting and posturing that is associated with severe dystonia in humans [24]. Importantly, EMG has been a powerful tool to test for abnormal muscle contractions in mouse models [24, 61]. Regardless of the exact phenotype, the dystonia rating scale, originally adapted from human patient exams, remains useful for mouse models [62]. The *Ptf1a*^*Cre/*+^*;Vglut2*^*flox/flox*^ mutant mice offered a genetically precise method of testing the contribution of cerebellar circuits, with the severe twisting postures, clear tremor, developmental onset, and the lack of overt neurodegeneration providing a means for dissecting the neural mechanisms that drive dystonia.

The *Ptf1a*^*Cre/*+^*;Vglut2*^*flox/flox*^ mutant mice have provided some key points for further consideration. (1) The developmental onset of the dystonia in this mouse indicates that the causative defects may derive from a dynamic set of structural and functional interactions long before the mature circuit is established. This idea is supported by the phenotype of a different mouse model in which granule cell neurogenesis is obstructed [46]. (2) Dystonia is co-morbid with many conditions. In ataxia, which is considered a “cerebellum disease,” the extent of co-occurrence is so common that algorithms have been created to aid in the diagnoses of ataxia-dystonia syndromes [63]. In our mouse model though, the use of EMG could be used to distinguish the co- and over-contractions from the abnormal muscle activity observed in tremor [41] and ataxia [36]. (3) Work from Slaughter and colleagues in the 1970s focusing on congenital athetosis reported a periodic burst firing of cerebellar neurons recorded in humans [64]. Similar defects were reported in the dt rat and confirmed in the cerebellar ouabain infusion model [19, 28]. Cerebellar nuclear neurons in the *Ptf1a*^*Cre/*+^*;Vglut2*^*flox/flox*^ mutant mice also exhibit a dramatic burst firing phenotype [31]. (4) Deep brain stimulation targeted to the GPi can have impressive effects, but interestingly, the benefits may be linked to a prominent effect on the cerebellum [65, 66]. In the *Ptf1a*^*Cre/*+^*;Vglut2*^*flox/flox*^ mice, stimulation of the interposed nucleus significantly improves mobility [31] (Fig. 5). Although the mechanism of how deep brain stimulation recovers circuit function in different motor diseases remains unclear [67], in our model, modulation of the burst output at the level of the cerebellar nuclei appears to be important. Perhaps specific abnormal signals such as the bursts could be targeted for recovery, an idea that is supported by the restoration of movement when ataxic mice with erratic cerebellar activity are fed an activator of Ca(2 +)-dependent K( +) channels called chlorzoxazone [68]. In the *Car8*^*wdl/wdl*^ mice, which have dystonia, ataxia, and tremor, chlorzoxazone recovers movement and corrects Purkinje cell firing by eliminating burst activity [45]. We postulate, therefore, that selective or designer drugs that target cellular level disease biomarkers could have beneficial outcomes as therapies.

There are also notable shortcomings of the *Ptf1a*^*Cre/*+^*;Vglut2*^*flox/flox*^ model. First, even though the dystonia is reliable (100% penetrance in mice with the correct alleles), robust, and easily quantifiable, the phenotype is not reversible. It would be remarkable to have an equivalent model in which the dystonia, and the associated cellular defects, could be eliminated on demand and at different time points during the animal’s life. One could then examine what aspects of the circuit maintain enough function for recovery, therefore unveiling additional therapeutic targets; whether specific circuits exhibit plasticity-related changes during dystonia, and an experimenter could carefully examine how circuits adapt during the onset and removal of dystonia. Second, even though the loss of climbing fiber to Purkinje cell function could be a feature in human dystonia (biallelic variants in *TSPOAP1*; [69]), it remains unclear how specific defects at this synapse relate to the many genetic forms of the disease, and specifically whether this circuit is a consistent target in the disease regardless of the initial insult. Importantly, though, we observed that at least some aspects of the phenotype, such as early postnatal Purkinje cell morphological and simple spike electrophysiological defects, do spontaneously recover as the animal matures [31]. Regardless of how the defect is generated in the different models, there is strong evidence that abnormal output of the cerebellar nuclear neurons, in this case with an enhanced burst quality of spike activity, is a primary feature of dystonia. Therefore, despite the limitations, this mouse model offers a multitude of mechanistic inroads for studying dystonia.

The design of strategies to target the cerebellum with noninvasive and invasive methods of brain stimulation has recently gained wide appreciation in the field [67]. Although the *Ptf1a*^*Cre/*+^*;Vglut2*^*flox/flox*^ mouse provided an ideal model to test the role of the interposed nucleus as a target for stimulation, it remains possible that the fastigial and dentate nuclei could also offer similar or even complimentary benefits. A patient with hemidystonia showed improved motor function after deep brain stimulation of the dentate nucleus [70]. More broadly, the dentate could be a powerful source of signals for repatterning connected regions, as indicated by electrical stimulation [71] and optogenetic stimulation [72] in rats and mice models of stroke, respectively. However, given the extensive excitatory and inhibitory output connectivity of the cerebellar nuclei [73, 74], not only are sub-nuclei specific circuits likely to be important, but the exact mode of communication and how signals are integrated at the target regions are almost certainly of major relevance to how effective a given stimulation paradigm will be. The further development of sophisticated genetic approaches that aim to manipulate the molecular, cellular, and functional properties of the dystonia circuit will enhance experimental possibilities and provide the means to address the many challenges of solving dystonia. In addition, the identification of new behavioral roles of the cerebellum that encompass motor and non-motor domains [75] and existing knowledge of cerebellar-related functional defects in human dystonia patients [76–78] highlight an exciting framework to more deeply test how cerebellar circuits impact dystonia. We propose that the complexity of behaviors observed across the different dystonias may be reflected within the extensive connectivity of the cerebellar system.
